# Equine Discomfort Ethogram

**DOI:** 10.3390/ani11020580

**Published:** 2021-02-23

**Authors:** Catherine Torcivia, Sue McDonnell

**Affiliations:** 1 Department of Clinical Studies, University of Pennsylvania School of Veterinary Medicine, 382 W Street Road, Kennett Square, PA 19382, USA; 2 Havemeyer Equine Behavior Lab and Clinic, University of Pennsylvania School of Veterinary Medicine, 382 W Street Road, Kennett Square, PA 19382, USA; suemcd@vet.upenn.edu

**Keywords:** behavior, horse, welfare, pain

## Abstract

**Simple Summary:**

Pain and discomfort behavior in horses tends to be especially subtle, and not readily or widely appreciated even by equine professionals, including many long-time horse keepers, trainers, and even by veterinarians, veterinary technicians, and care staff. Based on decades of evaluating the behavior of normal and physically uncomfortable horses in a referral hospital, as well as research context, we describe and illustrate a catalog of behaviors (ethogram) associated with equine physical discomfort. Our objective is to promote an unambiguous universal understanding of equine discomfort behaviors associated with various body systems and anatomic sources.

**Abstract:**

In recent years, there has been a growing interest in and need for a comprehensive ethogram of discomfort behavior of horses, particularly for use in recognizing physical discomfort in domestically managed horses. A clear understanding of the physical discomfort behavior of horses among caretakers, trainers, and professional health care personnel is important to animal welfare and caretaker safety. This is particularly relevant to pain management for hospitalized equine patients. Various pain scale rubrics have been published, typically incorporating only a few classically cited pain behaviors that, in many cases, are specific to a particular body system, anatomic location, or disease condition. A consistent challenge in using these rubrics in practice, and especially in research, is difficulty interpreting behaviors listed in various rubrics. The objective of this equine discomfort ethogram is to describe a relatively comprehensive catalog of behaviors associated with discomfort of various degrees and sources, with the goal of improving understanding and clarity of communication regarding equine discomfort and pain. An inventory of discomfort-related behaviors observed in horses has been compiled over 35 years of equine behavior research and clinical consulting to medical and surgical services at the University of Pennsylvania School of Veterinary Medicine’s equine hospital. This research and clinical work included systematic evaluation of thousands of hours of video-recordings, including many hundreds of normal, healthy horses, as well as hospitalized patients with various complaints and/or known medical, neurologic, or orthopedic conditions. Each of 73 ethogram entries is named, defined, and accompanied by a line drawing illustration. Links to online video recorded examples are provided, illustrating each behavior in one or more hospitalized equine patients. This ethogram, unambiguously describing equine discomfort behaviors, should advance welfare of horses by improving recognition of physical discomfort, whether for pain management of hospitalized horses or in routine husbandry.

## 1. Introduction

The recognition of physical discomfort in horses is important to ensuring adequate welfare, both for general husbandry and for veterinary care procedures. Traditional veterinary evaluation of pain in equine patients has relied heavily on objective physical measures (e.g., heart rate, respiratory rate), and to a lesser extent, endocrine measures (e.g., circulating levels of cortisol, ß-endorphins, catecholamines, or pro-inflammatory mediators), mostly in research applications [1,2]. None of these, alone or in combination, have been found to be definitive indicators of pain, due to multiple complicating factors, resulting in an inability to distinguish pain from other physiological or psychological stress [1,2]. In an effort to more accurately evaluate pain in horses, in recent decades, multiple composite pain scales have been designed to take into account both objective physical measures and classically cited, observable behaviors associated with discomfort [3,4,5]. Despite this progress, a recent review of these equine pain scoring systems emphasizes that there are still important shortcomings with the existing equine pain scales, specifically recognizing mild pain states [1]. One factor contributing to this may be the lack of detailed descriptions, images, or video examples of behaviors included in the scoring systems. In our experience, in practice, this ambiguity leads to considerable confusion resulting in poor recognition of discomfort. One exception is the Horse Grimace Scale (HGS) designed by Dalla Costa et al. [6], which includes detailed descriptions and photographs to illustrate various components of facial expressions associated with discomfort. To our knowledge, a comprehensive equine discomfort ethogram has not been published. Certain reports do describe some specific discomfort behaviors in post-operative equine patients following particular surgical procedures, for example, orthopedic surgery [7,8] or celiotomy [9]. An earlier review also grouped behavioral indicators of pain into categories based on body system affected [10]. However, there is still a need for a relatively comprehensive and well-illustrated equine discomfort ethogram that would be easily understood across various languages and horse care cultures.

The validity of any pain scoring system that includes behavioral indicators requires that (1) the developers clearly understand the various behaviors that may indicate discomfort in horses and (2) that users universally understand and are able to recognize those behaviors. The objectives of this report were to (1) unambiguously describe a relatively comprehensive list of behaviors that in decades of clinical experience we have observed to be associated with the physical discomfort of various degrees and sources in horses, providing both line drawing illustrations and video examples, and (2) for various clinical conditions, describe clusters of those behaviors we have commonly observed.

## 2. Background Details

### 2.1. Original Observations

The information presented in this ethogram was primarily collected by the senior author (SM) over 35 years of equine behavior research and clinical consulting at the University of Pennsylvania School of Veterinary Medicine New Bolton Center. This work included systematic evaluation of thousands of individual horses, each with a minimum of 24 h of continuous video recording. These included normal, healthy horses in research studies or in breeding programs, as well as hospitalized patients with various presenting complaints and/or known medical, neurologic, or orthopedic conditions. Patient behavior evaluations were performed primarily to assist hospital clinicians or referring veterinarians with their diagnostic process. Typically, this involved advising whether a change in behavior or performance appeared to more likely to reflect a psychological/behavioral problem and/or current physical discomfort [11]. In most cases, a source of physical discomfort was eventually diagnosed, providing feedback that enhanced the evaluator’s body of knowledge. During this work, an inventory of discomfort-related behaviors associated with various body systems and anatomic sources in horses was compiled. This work also often included evaluation of behavior before and after administration of analgesia. In these cases, if a behavior was suspected to reflect pain, and if it diminished with the administration of analgesia, the behavior could more confidently be judged to reflect pain. At times, clinicians also requested the author’s (SM) professional opinion concerning the level of discomfort or quality of life in patients with known painful conditions. These consults provided further information concerning types of discomfort behavior associated with various body systems and anatomic sources.

The standard behavior consultation performed for these services involves an analysis of a 24-h (or occasionally longer) continuous video-recorded sample of a horse in its stall. This method includes observation during periods without caretaker presence, which provides more detailed and complete information regarding discomfort behavior than direct observation [11]. Caretaker presence has been shown to disrupt ongoing discomfort behavior in horses [7]. Additionally, the ability to scan video in fast forward enhances the recognition of repetitive behaviors and postures that are typically more difficult to identify in shorter periods of direct observation.

Before 2017, the majority of these evaluations did not involve patients with orthopedic conditions requiring surgery. To ensure a comprehensive inventory of discomfort behaviors associated with a variety of orthopedic conditions, in 2017, the authors together evaluated a set of 60 hospitalized patients undergoing elective or emergency orthopedic surgical procedures (e.g., arthroscopy, fracture repairs, arthrodesis). For each horse, a minimum of 48 h of continuous video footage was obtained, including samples before and after surgery. Specific discomfort behaviors associated with various orthopedic conditions were cataloged in a manner similar to that of observations from the previous 35 years. All animal procedures for obtaining video recordings during the 2017 pain assessment study were approved by the University of Pennsylvania Institutional Animal Care and Use Committee (protocol #806321).

### 2.2. Behavior Inventory from the Literature

A search of veterinary, animal science, and equine behavior science literature (English language, from mid 20th century to the present) was undertaken to identify research reports and review articles or book chapters describing behaviors associated with physical discomfort in horses. Forty-five such sources [1,2,3,4,5,6,7,8,9,10,11,12,13,14,15,16,17,18,19,20,21,22,23,24,25,26,27,28,29,30,31,32,33,34,35,36,37,38,39,40,41,42,43,44,45] were reviewed to confirm agreement with our identified behaviors, as well as to identify any behavioral elements or sequences reported to reflect physical discomfort in horses that had not been identified in our clinical observations.

## 3. Ethogram

The resulting list of equine discomfort behaviors in this ethogram includes a total of 64 specific discomfort behaviors grouped into eight categories: posture and weight-bearing; limb and body movements; head, neck, mouth, and lip movements; attention to area; ear and tail movements; overall demeanor; altered eating or drinking; and vocalizations/audible sounds. Some behaviors have slight variations in form (e.g., stretching), resulting in a total of 73 entries (see Table 1, Table 2, Table 3, Table 4, Table 5, Table 6, Table 7 and Table 8). Each entry includes a name we believe to be commonly used in English, a word definition, a line drawing depiction, and (with one exception, *sipping water*) a link to a supplemental video file depicting one or more examples of equine patients displaying the behavior. Many, but not all, of these behaviors, have been mentioned in the literature that we reviewed. There were no additional behaviors described in the literature that we did not record in our clinical observations. In addition, Table S1 summarizes behaviors commonly associated with various anatomic sources of discomfort.

## 4. Comments

We propose that this ethogram provides a relatively complete catalog of behaviors that can be used as a reference to recognize discomfort in horses, both for general husbandry and for clinical veterinary assessments. Hopefully, it will also prove useful for research and for future pain scale development or refinement.

Recognition of discomfort in a prey species is particularly challenging. Horses have evolved to show little evidence of discomfort or disability in the presence of predators, including humans. This obviously can confound discomfort assessment [36]. In our clinical review of 24-h continuous video, the effect of this phenomenon has been conspicuous (11). In their pain assessments of horses following arthroscopic surgery, Price et al. [30] commented that patients in their study showed a reduced incidence of certain pain behaviors (e.g., restlessness, weight shifting) when observed directly, even when caretakers viewed from a distance, compared to remotely by video. Recently, we quantitatively evaluated this important effect in 20 hospitalized equine orthopedic patients [7]. In those patients, ongoing discomfort behaviors diminished or stopped altogether during caretaker visits for pain assessment, and resumed once the caretakers had departed. Those patients displayed a mean reduction of over 75% in the number of discomfort behaviors per minute when caretakers were present, and 30% of those patients stopped performing discomfort behaviors altogether during the caretaker visit. Due to this effect, composite pain scoring systems that require a period of direct observation likely underestimate discomfort behaviors. We, therefore, posit that, when assessing discomfort in horses, it is important to observe remotely. Regardless of how well-trained a caretaker may be in behavior observation, if discomfort behavior is interrupted by their presence, information regarding the horse’s condition is lost.

Brief periods of direct observation may also lead to misinterpretations of comfort. For example, stalled horses typically go through cycles of foraging and resting, often always standing in one particular area of the stall when resting. If not observing longer periods of continuous video, it may appear that a horse rarely moves from one area, when they are actually going through normal resting/foraging cycles, but always returning to the same area for standing rest. Similarly, if a caretaker happened to repeatedly visit during rest periods, they may erroneously conclude that the horse has a decreased appetite. For this reason, observing for longer periods of time via video recording, which can be viewed in fast forward, is invaluable in understanding the overall behavior pattern of a horse.

Behaviors that suddenly interrupt ongoing goal-directed behavior, such as foraging or resting, when the horse is otherwise calm, more clearly appear to represent acute discomfort. In our experience, deviation from, or apparent inability to perform, normal sequences of foraging and resting behavior, has been associated with increased levels of discomfort.

Our experience with video observation of such a large number of clinical cases, many with similar sources and types of diagnosed physical discomfort, has allowed us to appreciate that there appear to be individual differences among horses in the expression and the particular combination of various discomfort behaviors for any particular diagnosed condition. Ijichi et al. [46] recently explored the association of pain expression and corresponding severity of musculoskeletal lesion in horses with owner-assessed personality factors. An important finding in that work was that the degree of clinical lameness was not a reliable indicator of the severity of tissue damage as diagnosed on ultrasound or radiographic imaging. The conclusion of their preliminary study was that individual variation in the expression of pain may be associated with certain personality factors.

Many of the discomfort behaviors included in this ethogram can be expressed with slight variation in form. For example, as described in Table 2e, kicking out or back can be performed in several distinct forms, varying in height and degree of extension of the limb or limbs. In this ethogram, our illustration depicts just one common form. We expect that variations will be easily recognizable as that behavior. Experience observing horses over time will likely increase the observer’s knowledge of the possible variations of particular behaviors.

We would not consider a single occurrence of any one behavior to be conclusive evidence of discomfort. When viewing video of a horse to assess comfort level, in most instances, we consider the first occurrence of a potential discomfort behavior as an indicator to continue watching for repetitions of that particular behavior. Before making a judgment about the causes of a behavior, or what a specific behavior might indicate regarding discomfort, it is important to be sure that it was not an isolated event with an alternate explanation. Repetitive clusters of behaviors, including one or more non-specific discomfort behaviors (e.g., *moving/focusing ears caudally, swishing/flicking tail, rotational shaking head or whole body, restlessness/ill-at-ease*), are also helpful in confidently judging that a behavior reflects physical discomfort. For example, if one or more episodes of stomping are observed without any other indicators of discomfort, one might be more suspicious of superficial irritation, such as a biting insect. In our experience, additional specific indicators of limb pain (e.g., *non-physiologic locomotion, shifting weight/resting limb, pointing*) or multiple non-specific indicators of discomfort, typically increase confidence that these behaviors reflect pain. Similarly, particular combinations of temporally-associated behaviors often provide insight as to the anatomic source of discomfort. (see supplementary Table S1: Discomfort Behaviors Commonly Associated with Various Anatomic Sources https://doi.org/10.5281/zenodo.4537909 (accessed on 18 February 2021)).

This ethogram is based on observations of stalled horses at rest. It does not necessarily address discomfort behaviors that may be observed in horses during work. For a recently published pain ethogram for horses while being ridden, see Dyson et al. 2018 [47]. Further, and importantly, our ethogram is not meant to be independently diagnostic, but rather to provide additional detailed information to veterinary professionals. Should routine caretakers or health care professionals observe behavior suggesting discomfort, further veterinary diagnostics are indicated.

Finally, within the context of various horse behavior courses and summer research student training, we have had the opportunity to introduce approximately 50 graduate students, veterinary students, veterinarians, horse owners, and trainers to this method of evaluating discomfort in horses using video recorded behavior of horses in stalls. Our informal measures of training time suggest the rapid acquisition of the skill. Following a review of earlier versions of this ethogram, reliable recognition of these behaviors has been almost immediate. The typical time for a new observer to reach an acceptable inter-observer agreement with highly experienced observers has been less than 3 h of experience with the technique (unpublished observations).

## 5. Conclusions

This ethogram, unambiguously describing equine discomfort behaviors, promises to advance welfare of horses by improving recognition of physical discomfort, whether for pain management of hospitalized horses or in everyday husbandry.

**Table 1:** Posture and weight-bearing.

*a. Non-physiologic Locomotion* 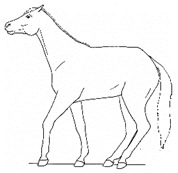	Lameness, including altered stride, impact, and weight-bearing. May include altered limb placement, head, and neck movements that suggest off-loading, and/or limited range of motion of a limb. Video S2 https://doi.org/10.5281/zenodo.4537915 (accessed on 18 February 2021)
*b. Shifting Weight/Resting Limb* 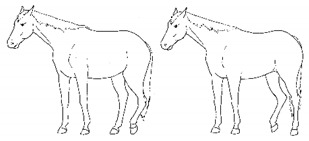	Frequent shifting of the primary weight-bearing limb or limbs. The frequency of abnormal weight shifting is typically greater during the transition to standing rest, as if the horse is having difficulty finding a comfortable resting position. Video S3 https://doi.org/10.5281/zenodo.4537972 (accessed on 18 February 2021)
*c. Pointing* 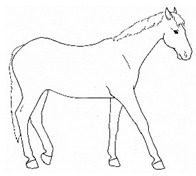	Consistent extension of one forelimb, reducing weight-bearing on that limb. Pointing is often most apparent during periods of standing rest, but can also occur during foraging, when one forelimb is consistently placed cranially, while the other is placed caudally and under the body (healthy comfortable horses typically either stand squarely on forelimbs or alternate forelimb placement as they move while foraging). Video S4 https://doi.org/10.5281/zenodo.4537978 (accessed on 18 February 2021)
*d. Prolonged Resting of Limb* 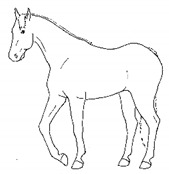	Flexing a fore or hindlimb, resting the toe of the hoof on the substrate. Hindlimb resting is a normal behavior, particularly during standing rest. Prolonged resting of a particular limb, especially when standing alert or foraging, may indicate discomfort. Video S5 https://doi.org/10.5281/zenodo.4538011 (accessed on 18 February 2021)
*e. Cross-legged Resting of Limb* 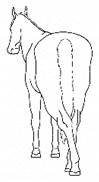	Resting a limb (usually hind) crossed slightly behind or in front of the opposite limb. Video S6 https://doi.org/10.5281/zenodo.4538028 (accessed on 18 February 2021)
*f. Camping Under* 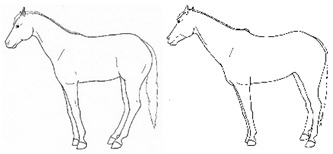	Standing with fore and/or hindlimbs bilaterally positioned underneath the abdomen, sometimes associated with a hunched back posture. Weight-bearing is greater on the camped under limbs or, in the case of camping under on both fore and hindlimbs, may be associated with relieving weight-bearing of the back. Video S7 https://doi.org/10.5281/zenodo.4538039 (accessed on 18 February 2021)
*g. Dragging a Limb* 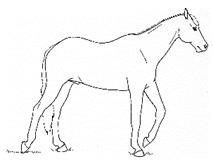	Partially flexing a limb such that the toe scrapes along or slightly above the substrate when moving. Video S8 https://doi.org/10.5281/zenodo.4539062 (accessed on 18 February 2021)
*h. Dangling a Limb* 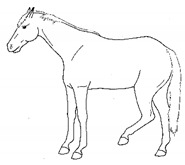	Flexing and hanging a limb above the substrate for several seconds or longer, often in a series, momentarily resting the tow lightly on the substrate between lifts. Video S9 https://doi.org/10.5281/zenodo.4538101 (accessed on 18 February 2021)
*i. Base Narrow or Wide* 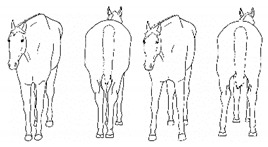	Standing or moving with fore and/or hindlimbs placed either more medially or laterally than is normal for the horse’s conformation. Video S10 https://doi.org/10.5281/zenodo.4538117 (accessed on 18 February 2021)
*j. Low Head Carriage* 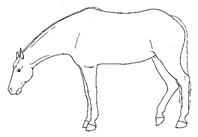	Moving or standing with the neck below horizontal. In deep standing rest, it is normal for the head and neck to drop below horizontal. *Low head carriage* is often associated with *dull overall demeanor* or exhaustion. Video S11 https://doi.org/10.5281/zenodo.4538125 (accessed on 18 February 2021)
*k. Tucked Up Abdomen* 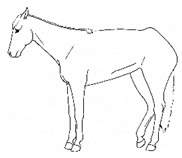	Tensing of the abdominal muscles, hollowing out the flank, sometimes with a hunched back. Reduced ingesta and dehydration may contribute to the hollow appearance (also known as “drawn up” or “sucked up”). Video S12 https://doi.org/10.5281/zenodo.4538135 (accessed on 18 February 2021)
*l. Leaning Against Objects* 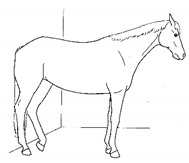	Supporting weight and/or stabilizing balance against a wall or fence, usually during standing rest. Video S13 https://doi.org/10.5281/zenodo.4539088 (accessed on 18 February 2021)
*m. Atypical Recumbency* 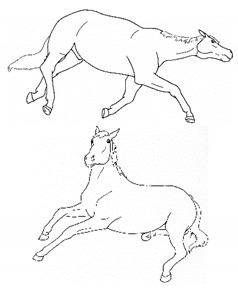	Prolonged or frequent interrupted recumbent rests and/or increased recumbent resting time budget. *Atypical recumbency* may occur during circumstances when the horse would not normally lie down, and the horse may lie in full lateral recumbency without sleeping. *Atypical recumbency* may include *sighing* and/or *groaning*, tense facial muscles, gaping mouth, *lip quivering/wincing*, *teeth grinding*, and/or atypical limb placement. For healthy horses, typical individual recumbency durations range from 15 to 40 min. Prolonged recumbency may be related to *difficulty rising*, discomfort standing, or exhaustion. Videos S14 and S15 https://doi.org/10.5281/zenodo.4539094 (accessed on 18 February 2021)
*n. Difficulty Rising* 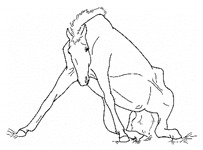	Failing to rise gracefully, requiring increased effort and/or attempts to rise. Video S16 https://doi.org/10.5281/zenodo.4539120 (accessed on 18 February 2021)
*o. Urination Posture and Effort Without Stream* 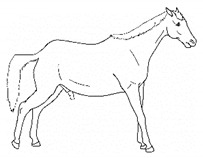	Posturing to urinate, often repeatedly for extended durations, with apparent effort, but without a normal stream of urine. Often accompanied by *groaning, swishing, or slapping tail, ears focused caudally, looking* caudally, and/or *kicking up toward abdomen.* Video S17 https://doi.org/10.5281/zenodo.4539122 (accessed on 18 February 2021)
*p. Parking Out* 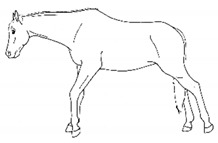	Standing with forelimbs positioned forward and hindlimbs positioned behind a normal “squared up” stance. Video S18 and Photo S19 https://doi.org/10.5281/zenodo.4539206 (accessed on 18 February 2021)
*q. Straining to Defecate* 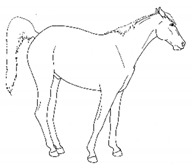	Exerting greater than the normal abdominal effort to pass feces. Often accompanied by *groaning, swishing/flicking tail, slapping tail against perineum, ears focused caudally, looking caudally, and/or kicking up toward abdomen.* Video S20 https://doi.org/10.5281/zenodo.4539216 (accessed on 18 February 2021)

**Table 2:** Limb and body movements.

*a. Stepping in Place* 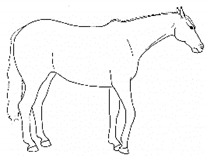	Repeated flexing of a limb, briefly relieving weight-bearing on that limb. The toe may touch or be held slightly above the substrate for up to several seconds, before the hoof is lightly placed down again. Stepping often occurs in a series of several rhythmic steps at about 1 s intervals, with a pause between series. Video S21 https://doi.org/10.5281/zenodo.4540099 (accessed on 18 February 2021)
*b. Lifting/Holding Limb Up* 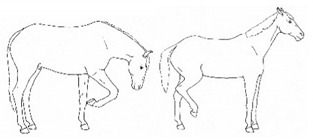	Flexing a forelimb, lifting the hoof off the substrate (usually 20 cm or more), and then placing it back down, similar to that seen in response to cutaneous irritation, e.g., insects. Video S22 https://doi.org/10.5281/zenodo.4540101 (accessed on 18 February 2021)
*c. Pawing* 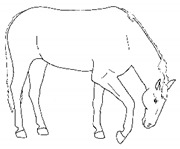	Reaching a forelimb cranially and dragging the hoof along or above the substrate while sweeping caudally, often in rhythmic series. This behavior is similar to that which often occurs within the context of foraging or drinking (particularly when thwarted, or in anticipation). Video S23 https://doi.org/10.5281/zenodo.4540104 (accessed on 18 February 2021)
*d. Stomping* 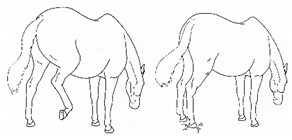	Suddenly flexing and then extending a limb, sharply striking the hoof against the substrate, similar to that seen in response to cutaneous irritation, e.g., insects. Video S24 https://doi.org/10.5281/zenodo.4540107 (accessed on 18 February 2021)
*e. Kicking Out or Back* 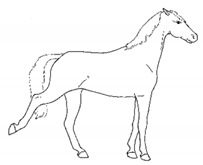	Lifting and extending one or both hindlimbs caudally, either straight back or sometimes arcing laterally. Video S25 https://doi.org/10.5281/zenodo.4540109 (accessed on 18 February 2021)
*f. Kicking Up Toward Abdomen* 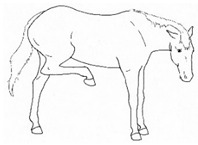	Flexing a hindlimb, directing the hoof or stifle toward the abdomen, often arcing laterally as the hoof returns toward the substrate, often similar to that seen in response to cutaneous irritation, e.g., insects. Video S26 https://doi.org/10.5281/zenodo.4540113 (accessed on 18 February 2021)
*g. Romping/Bucking* 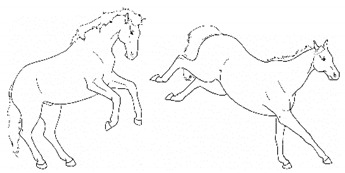	Alternate rearing and kicking out, often repeatedly in rapid succession. Romping and bucking may indicate an acutely painful event that appears to startle the horse; or alternatively, an outburst reflecting frustration with an inability to get relief from discomfort. Video S27 https://doi.org/10.5281/zenodo.4540115 (accessed on 18 February 2021)
*h. Rolling* 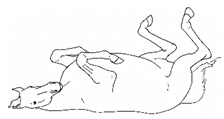	Lying down to sternal recumbency and then rotating from sternal to lateral and then dorsal recumbency, sometimes over from one side to the other. Video S28 https://doi.org/10.5281/zenodo.4540117 (accessed on 18 February 2021)
*i. Backing* 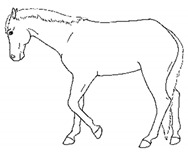	Walking backwards tentatively, as if attempting to retreat from discomfort. Video S29 https://doi.org/10.5281/zenodo.4540123 (accessed on 18 February 2021)
*j. Limb Trembling* 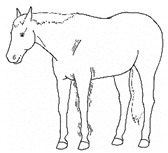	Rhythmic muscle tremors. *Limb trembling* may, in some instances, reflect muscle exhaustion or effort to relieve weight-bearing of a limb. With reduced weight-bearing, the entire limb may quiver with the trembling. Video S30 https://doi.org/10.5281/zenodo.4540125 (accessed on 18 February 2021)
*k. Flinching* 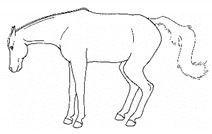	Sudden reflexive contraction of muscles. *Flinching* may indicate an acutely painful event that startles the horse, causing sudden escape movement and/or loss of balance. Video S31 https://doi.org/10.5281/zenodo.4540528 (accessed on 18 February 2021)
*l. Stretching* 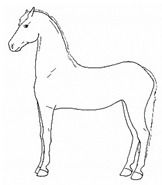	i. *Head High* Raising and pulling head caudally, with the back curved ventrally. Head may be held in different positions ranging from horizontal to curled ventrally with muzzle drawn toward the chest. Video S32 https://doi.org/10.5281/zenodo.4540532 (accessed on 18 February 2021)
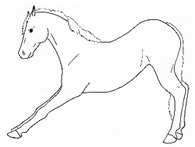	ii. *Deep Abdominal* Extending forelimbs cranially, shifting weight onto hindlimbs with the shoulders lowered toward ground, and the back curved ventrally. Video S33 https://doi.org/10.5281/zenodo.4540538 (accessed on 18 February 2021)
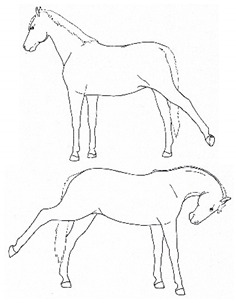	iii. *Hindlimb Extension* Extending a hindlimb caudally, occasionally with a *neck curl stretch*, and sometimes with the back curved ventrally. Video S34 https://doi.org/10.5281/zenodo.4540542 (accessed on 18 February 2021)
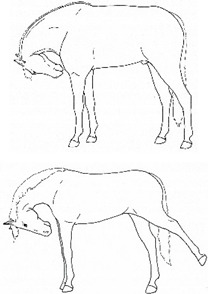	iv. *Neck Curl* Arching the neck, with muzzle to chest, back arched dorsally, usually with a *hindlimb extension stretch.* Video S35 https://doi.org/10.5281/zenodo.4540548 (accessed on 18 February 2021)

**Table 3:** Head, neck, mouth, and lip movements.

*a. Rotational Head or Whole Body Shaking* 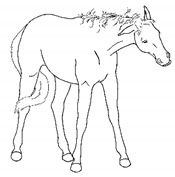	Rapid, rhythmic rotation of the whole body or just the head and neck along the long axis. This is similar to shaking in response to cutaneous irritation around the head, neck, or body, e.g., insects. *Rotational head or whole body shaking* is often associated with bearing weight on an affected limb or another behavior, such as *lifting* or *kicking* a limb. May occur during prolonged periods of unrelieved discomfort, often during episodes of *fidgeting*. Video S36 https://doi.org/10.5281/zenodo.4540550 (accessed on 18 February 2021)
*b. Frustration Head Tossing* 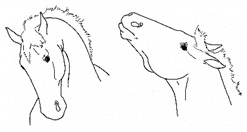	Quick rotational toss of the head, similar to a head threat. Video S37 https://doi.org/10.5281/zenodo.4540554 (accessed on 18 February 2021)
*c. Head Bobbing* 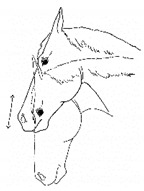	Repetitive nodding of head and neck. Not usually as rhythmic as with a stereotypy. Often appears to reflect frustration with persistent discomfort, particularly when the horse seems unable to find a comfortable posture. Video S38 https://doi.org/10.5281/zenodo.4540559 (accessed on 18 February 2021)
*d. Nose Tossing/Flipping* 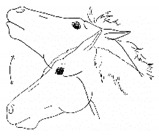	Flicking upward extension of the head and neck. Not usually rhythmic, as with a stereotypy. Often appears to reflect frustration with persistent discomfort, particularly when the horse seems unable to find a comfortable posture. Video S39 https://doi.org/10.5281/zenodo.4540563 (accessed on 18 February 2021)
*e. Abbreviated Weaving* 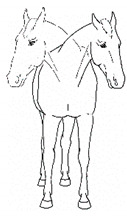	Rhythmic side-to-side swaying of the head and neck. Typically does not include animated weight shifting and alternate flexing, lifting, and/or lateral movement of forelimbs, as is common with the stereotypy form of weaving. Video S40 https://doi.org/10.5281/zenodo.4540565 (accessed on 18 February 2021)
*f. Sympathetic Surge Resolution Signs: Extending Tongue, Licking, Chewing, Itching* 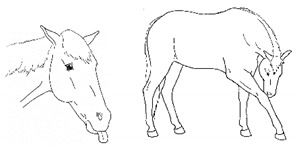	Cluster of autonomic responses following an acute sympathetic surge, including salivation (leading to chewing movements, swallowing, tongue extensions) and/or *autogrooming* (typically rubbing face against forelimb). Video S41 https://doi.org/10.5281/zenodo.4541367 (accessed on 18 February 2021)
*g. Frequent Yawning Bouts* 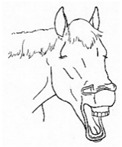	Frequent bouts of yawning, often with a greater number of yawns per bout than is characteristic (greater than 3 to 5). Often associated with changes in sympathetic tone, so may occur intermittently with lip licking, chewing, tongue extensions characteristic of *sympathetic surge resolution signs.* Video S42 https://doi.org/10.5281/zenodo.4541373 (accessed on 18 February 2021)
*h. Spontaneous Flehmen Response* 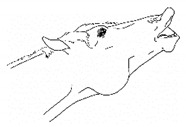	Lifting of the head with curling of the upper lip, drawing nasal fluids into the vomeronasal organ. *Flehmen* is a normal response within the context of sniffing pungent fluids, typically in a social olfactory context, but with discomfort, *flehmen* may occur out of that context. Video S43 https://doi.org/10.5281/zenodo.4541379 (accessed on 18 February 2021)
*i. Lip Quivering/Wincing* 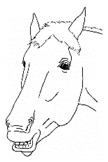	Involuntary movements (twitching) of the lips and nares, often with relaxation (drooping) of the lower lip. Video S44 https://doi.org/10.5281/zenodo.4541385 (accessed on 18 February 2021)
*j. Tilting Head* 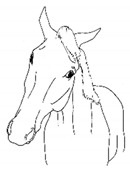	Holding or cocking the head to one side, such that the sagittal plane is off vertical. Video S45 https://doi.org/10.5281/zenodo.4541387 (accessed on 18 February 2021)

**Table 4:** Attention to an area.

*a. Looking* 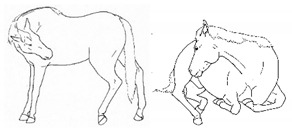	Glancing or gazing at a particular area of the body. Video S46 https://doi.org/10.5281/zenodo.4541390 (accessed on 18 February 2021)
*b. Swatting* 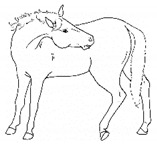	Swinging the head and neck, batting at a particular area of the body, similar to that seen in response to cutaneous irritation, e.g., insects. Video S47 https://doi.org/10.5281/zenodo.4541394 (accessed on 18 February 2021)
*c. Autogrooming* 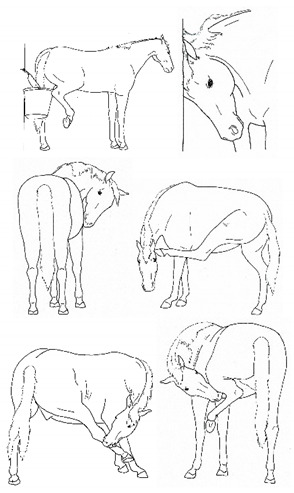	Nibbling, nuzzling, and/or biting at an area of the body, or rubbing one part of the body to another or against an object, similar to that seen in response to cutaneous irritation, e.g., insects. Video S48 https://doi.org/10.5281/zenodo.4541398 (accessed on 18 February 2021)

**Table 5:** Ear and tail movements.

*a. Moving/Focusing Ears Caudally* 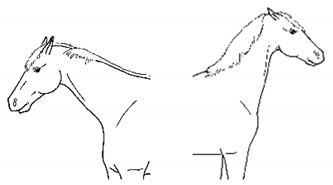	Rotating ears to focus caudally, or laying ears back against neck. Video S49 https://doi.org/10.5281/zenodo.4541402 (accessed on 18 February 2021)
*b. Swishing/Flicking Tail* 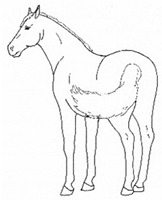	Moving tail suddenly from side to side, similar to that seen in response to cutaneous irritation, e.g., insects. Video S50 https://doi.org/10.5281/zenodo.4541408 (accessed on 18 February 2021)
*c. Slapping Tail Against Perineum* 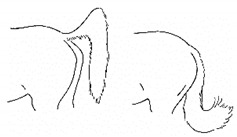	Sudden lifting and smacking of the tail against the perineum. Video S51 https://doi.org/10.5281/zenodo.4541736 (accessed on 18 February 2021)
*d. Lifting/Holding Tail Off Perineum* 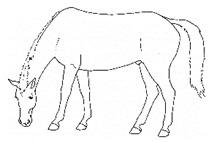	Raising the tail dorsally off the perineum for a few seconds or more. Video S52 https://doi.org/10.5281/zenodo.4541740 (accessed on 18 February 2021)

**Table 6:** Overall demeanor.

*a. Dull Expression/Depressed Demeanor* 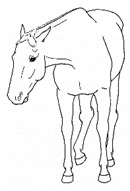	Less responsive to the environment, often with “zoned out,” worried, or glassy-eyed staring facial expression. Video S53 https://doi.org/10.5281/zenodo.4541763 (accessed on 18 February 2021)
*b. Guarding* 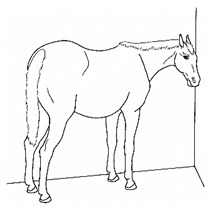	Especially cautious movement and retreat from potential disturbance or “cowering” submissive appearance as if threatened by humans or other horses. The horse may stand in the back of the stall or uncharacteristically not approach a person who enters. They may stand with muscles tensed and seem unwilling to move even when encouraged. Video S54 https://doi.org/10.5281/zenodo.4541765 (accessed on 18 February 2021)
*c. Conservative Movement* 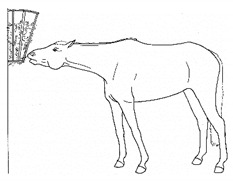	Less than typical movement about the stall, with apparent hesitation to walk (e.g., reaching with head and neck rather than stepping forward). Video S55 https://doi.org/10.5281/zenodo.4541767 (accessed on 18 February 2021)
*d. Uncharacteristic Aggression* 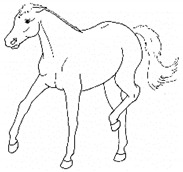	Increased irritability/aggression (biting, kicking, barging) toward people or other animals. Similar to *hyper-responsiveness*, a horse under increased stress from discomfort may display changes in demeanor that manifest as increased aggression. These horses may be generally sour or unexpectedly lash out at people or other animals. Video S56 https://doi.org/10.5281/zenodo.4541773 (accessed on 18 February 2021)
*e. Hyper-Responsive/Startle Prone* 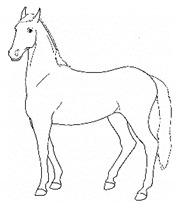	Lower threshold and more animated reaction to environmental stimuli. *Hyper-responsiveness* may indicate a low tolerance for stimuli, due to an increased stress level associated with discomfort. Palpation of the painful area may also illicit an uncharacteristically strong reaction. Video S57 https://doi.org/10.5281/zenodo.4543427 (accessed on 18 February 2021)
*f. Restlessness/Ill-at-ease*	i. *Changing Activities Frequently* Changing major activity (foraging, standing rest, standing alert) more frequently than expected, as often as from minute-to-minute, with other signs of discomfort. Video S58 (in 4x time) https://doi.org/10.5281/zenodo.4543432 (accessed on 18 February 2021)
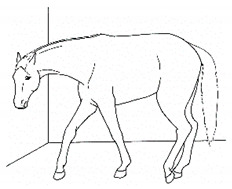	ii. *Circling/Pacing* Walking in circles or back and forth along a perimeter. In cases of mild to moderate discomfort, *circling/pacing* may interrupt ongoing behaviors in a manner that suggests the horse cannot find a comfortable standing position. Video S59 https://doi.org/10.5281/zenodo.4543444 (accessed on 18 February 2021)
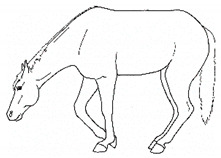	iii. *Abandoning Recumbency or Elimination Attempt* Repeated posturing as if intending to lie down, urinate, or defecate, that appears interrupted by discomfort (accompanied by, for example, *swishing/flicking tail, focusing ears caudally, rotational shaking head or whole body*). For example, attempts at recumbency during which the horse appears to commence lying down, (circle, paw the substrate, with head down and buckling at the knees), but then returns to standing, as if reluctant or uncomfortable lying down. Video S60 https://doi.org/10.5281/zenodo.4543446 (accessed on 18 February 2021)
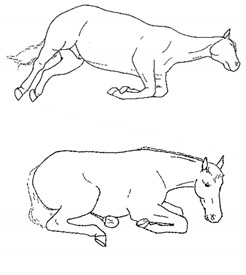	iv. *Frequent Repositioning During Recumbency* During recumbent rest, frequent alternating between sternal and lateral position, and/or repositioning of limbs or head and neck as if trying to find a comfortable position. Video S61 https://doi.org/10.5281/zenodo.4543448 (accessed on 18 February 2021)
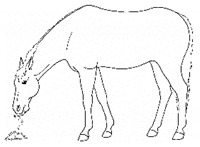	v. *Nibbling Aimlessly* Reduced interest in hay or feed, but continued nominal foraging gestures, often directed at non-food objects. The horse may have access to hay or feed, but instead picks around in bedding or only nibbles small bites alternately with other activities. The horse may nibble or lick at walls, feed or water containers, or other non-food objects. Video S62 https://doi.org/10.5281/zenodo.4543452 (accessed on 18 February 2021)
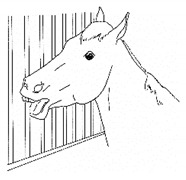	vi. *Fidgeting* Biting, mouthing, and/or rubbing against objects (e.g., stall walls, feed/water containers). These responses often reflect frustration with the inability to find a comfortable posture or relief from prolonged discomfort. Video S63 https://doi.org/10.5281/zenodo.4543456 (accessed on 18 February 2021)
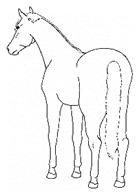	vii. *Intense Distantly-Focused Staring* Distant gazing in a fixed direction for a prolonged period, often with a glassy-eyed, tense, worried expression. Video S64 https://doi.org/10.5281/zenodo.4545919 (accessed on 18 February 2021)

**Table 7:** Altered eating or drinking.

*a. Sipping Water* 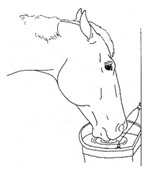	Drinking smaller than the typical volume of water, often with a tentative approach and expression of hesitation or discomfort
*b. Quidding* 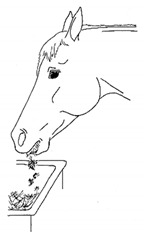	Dropping strands or partially chewed clumps of forage or grain from the mouth while eating. Video S65 https://doi.org/10.5281/zenodo.4545923 (accessed on 18 February 2021)
*c. Atypical Jaw Motion* 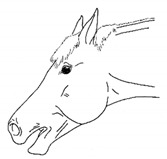	Conservative or atypical jaw movements, for example, when foraging, chewing, or yawning. Video S66 https://doi.org/10.5281/zenodo.4545933 (accessed on 18 February 2021)
*d. Disinterest in Food or Water* 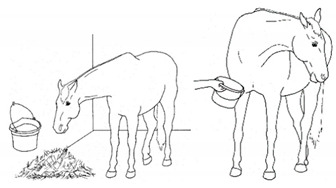	Reduced or no interest in palatable forage, grain, or water. Video S67 https://doi.org/10.5281/zenodo.4545947 (accessed on 18 February 2021)

**Table 8:** Vocalizations/audible sounds.

*a. Sighing*	Emitting an audible long exhalation following a deep inhalation.
*b. Snorting*	Emitting an audible sudden forced exhalation through the nares.
*c. Whining*	Emitting a long, high-pitched vocalization.
*d. Groaning*	Emitting a long, low-pitched, sometimes raspy, vocalization.
*e. Grunting*	Emitting a short, sharp, low-pitched vocalization.
*f. Squealing*	Emitting a short, sharp, high-pitched vocalization.
*g. Screaming/Calling*	Emitting a long loud whinny vocalization, typical of calling to locate distant herd mates.
*h. Teeth Grinding*	Moving tightly clenched jaws back and forth and grating the upper and lower molars, resulting in a crunching, scraping sound.

Videos S68–S75 https://doi.org/10.5281/zenodo.4545955 (accessed on 18 February 2021)

## Supplementary Materials

In addition to links to individual files which appear in each Table 1, Table 2, Table 3, Table 4, Table 5, Table 6, Table 7 and Table 8 entry, supplementary Table S1 and all video files for Table 1, Table 2, Table 3, Table 4, Table 5, Table 6, Table 7 and Table 8 are available together in one zip folder for download at: https://zenodo.org/badge/DOI/10.5281/zenodo.4477080.svg (accessed on 18 February 2021).
